# Inactivation of *farR* Causes High Rhodomyrtone Resistance and Increased Pathogenicity in *Staphylococcus aureus*

**DOI:** 10.3389/fmicb.2019.01157

**Published:** 2019-05-28

**Authors:** Minh-Thu Nguyen, Jongkon Saising, Paula Maria Tribelli, Mulugeta Nega, Seydina M. Diene, Patrice François, Jacques Schrenzel, Cathrin Spröer, Boyke Bunk, Patrick Ebner, Tobias Hertlein, Nimerta Kumari, Thomas Härtner, Dorothee Wistuba, Supayang P. Voravuthikunchai, Ulrike Mäder, Knut Ohlsen, Friedrich Götz

**Affiliations:** ^1^Microbial Genetics, Interfaculty Institute of Microbiology and Infection Medicine Tübingen (IMIT), University of Tübingen, Tübingen, Germany; ^2^Federal Regulatory Agency for Vaccines and Biomedicines, Paul Ehrlich Institute, Langen, Germany; ^3^School of Health Science, Mae Fah Luang University, Chiang Rai, Thailand; ^4^Departamento de Química Biológica, Facultad de Ciencias Exactas y Naturales – Universidad de Buenos Aires, IQUIBICEN-CONICET, Buenos Aires, Argentina; ^5^Genomic Research Laboratory, Service of Infectious Diseases, Geneva University Hospitals, Geneva, Switzerland; ^6^Leibniz Institute DSMZ-German Collection of Microorganisms and Cell Cultures, Braunschweig, Germany; ^7^Institute of Molecular Infection Biology, University of Würzburg, Würzburg, Germany; ^8^Microbiology/Biotechnology, Interfaculty Institute of Microbiology and Infection Medicine Tübingen (IMIT), University of Tübingen, Tübingen, Germany; ^9^Institute for Organic Chemistry, University of Tübingen, Tübingen, Germany; ^10^Department of Microbiology, Natural Product Centre of Excellence, Prince of Songkla University, Hat Yai, Thailand; ^11^Interfaculty Institute for Genetics and Functional Genomics, University Medicine Greifswald, Greifswald, Germany

**Keywords:** antibiotic, Gram-positive bacteria, rhodomyrtone, *Staphylococcus*, membrane active

## Abstract

Rhodomyrtone (Rom) is an acylphloroglucinol antibiotic originally isolated from leaves of *Rhodomyrtus tomentosa*. Rom targets the bacterial membrane and is active against a wide range of Gram-positive bacteria but the exact mode of action remains obscure. Here we isolated and characterized a spontaneous Rom-resistant mutant from the model strain *Staphylococcus aureus* HG001 (Rom^R^) to learn more about the resistance mechanism. We showed that Rom-resistance is based on a single point mutation in the coding region of *farR* [regulator of fatty acid (FA) resistance] that causes an amino acid change from Cys to Arg at position 116 in FarR, that affects FarR activity. Comparative transcriptome analysis revealed that mutated *farR* affects transcription of many genes in distinct pathways. FarR represses for example the expression of its own gene (*farR*), its flanking gene *farE* (effector of FA resistance), and other global regulators such as *agr* and *sarA*. All these genes were consequently upregulated in the Rom^R^ clone. Particularly the upregulation of *agr* and *sarA* leads to increased expression of virulence genes rendering the Rom^R^ clone more cytotoxic and more pathogenic in a mouse infection model. The Rom-resistance is largely due to the de-repression of *farE*. FarE is described as an efflux pump for linoleic and arachidonic acids. We observed an increased release of lipids in the Rom^R^ clone compared to its parental strain HG001. If *farE* is deleted in the Rom^R^ clone, or, if native *farR* is expressed in the Rom^R^ strain, the corresponding strains become hypersensitive to Rom. Overall, we show here that the high Rom-resistance is mediated by overexpression of *farE* in the Rom^R^ clone, that FarR is an important regulator, and that the point mutation in *farR* (Rom^R^ clone) makes the clone hyper-virulent.

## Introduction

Rhodomyrtone (Rom) is an antibiotic originally isolated from plant extracts of *Myrtaceae* species. The structural analysis showed that it belongs to the acylphloroglucinol class (Dachriyanus et al., 2002). Rom has antibiotic activity against a number of pathogenic Gram-positive bacterial species such as *Bacillus cereus*, *Enterococcus faecalis*, *Propionibacterium acnes*, *Staphylococcus aureus*, *Streptococcus pneumoniae*, or *Streptococcus pyogenes* (Saising et al., 2008, 2014; Voravuthikunchai et al., 2010; Limsuwan et al., 2011; Saising and Voravuthikunchai, 2012). The MIC values range from 0.1 to 2.0 μg/ml (Limsuwan et al., 2011).

Despite its antibacterial activity on a broad spectrum of bacteria, the exact MOA of Rom is still unknown. In a proteomic and a transcriptomic study in *S. aureus*, pleiotropic effects have been noted without clear correlation to a preferentially targeted cell structure or metabolic pathway (Sianglum et al., 2011, 2012). Rom does not address any of the classical antibiotic targets such as peptidoglycan biosynthesis, DNA-replication, translation, or transcription, but targets the cell membrane by causing a strong dissipation of the membrane potential and release of ATP and cytoplasmic proteins suggesting that Rom causes severe membrane disruption (Saising et al., 2018). Surprisingly, Rom does not significantly inhibit the oxygen consumption in *S. aureus* and does not form classical pores. However, being uncharged and devoid of a particular amphipathic structure, Rom does not seem to be a typical membrane-inserting molecule, but it causes formation of large membrane invaginations and it is transiently binding to phospholipid head groups (Saeloh et al., 2018).

Interestingly, the addition of certain FAs (pentadecylic acid, palmitic acid, and stearic acid) to the medium could counteract the antimicrobial activity (Saising et al., 2018). Although Rom does normally not inhibit Gram-negative bacteria, it has been shown that in the presence of PMBN (polymyxin B non-apeptide), MIC values for Rom in *Escherichia coli* dropped from >64 to approximately 1.0 μg/ml (Saising et al., 2018), indicating that Rom would be active against Gram-negative bacteria if Rom has a chance to penetrate the outer membrane.

At higher doses Rom is cytotoxic to different mammalian cells, and triggers in erythrocytes the translocation of phosphatidyl serine (PS) to the cell surface causing eryptosis, a suicidal erythrocyte death that is characterized by cell shrinkage and phospholipid scrambling in the cell membrane (Saising et al., 2018).

A number of semisynthetic Rom-derivatives have been synthetized, which showed less or similar activity as Rom (Leejae et al., 2012). It was also possible to completely synthesize Rom and its isomer rhodomyrtosone B, which showed a slightly higher activity than Rom (Morkunas et al., 2013). There were two alternative routes for Rom synthesis developed; one route is proposed as a possible pathway for Rom synthesis in plants (Morkunas and Maier, 2015). In a mouse skin infection model against MRSA it has been shown that rhodomyrtosone B prevents skin ulcer formation and shows a lower incidence of infection-induced morbidity; its activity was comparable to vancomycin (Zhao et al., 2018).

Here, we isolated a highly Rom-resistant mutant (Rom^R^) in *S. aureus*, which was due to a single point mutation in the *farR* gene. FarR was described as the transcriptional regulator of *farE* encoding an efflux pump for linoleic and arachidonic acids (Alnaseri et al., 2015). Here, we show that FarR is an important regulator that not only represses the expression of *farE* but also of various toxin genes; consequently, by inactivating *farR* these genes are de-repressed, which explains the increased pathogenicity of the Rom^R^ clone.

## Materials and Methods

### Bacterial Strains and Culture Conditions

Bacterial strains and plasmids are listed in Table 1. Unless stated otherwise, bacteria were grown aerobically in tryptic soy broth (TSB, Fluka) at 37°C under continuous shaking, or in basic medium [BM, 1% (w/v) casein peptone, 0.5% (w/v) yeast extract, 0.5% (w/v) NaCl, 0.1% (w/v) K2HPO4, 0.1% (w/v) glucose, pH 7.2]. Antibiotics were added when appropriate in the following concentrations: 100 μg ml^−1^ ampicillin in the case of *E. coli*, and 10 μg ml^−1^ chloramphenicol in case of *S. aureus* caryring pCX plasmid.

**Table 1:** Strains and plasmids used in this study.

Strains		References
***Staphylococcus aureus* NCTC 8325 derivatives**		
Original name	Short name	
HG001	HG001	Herbert et al., 2010
Rom^R^	Rom^R^	This study (point mutation in *farR*)
Rom^R^ Δ*farE*	Δ*far*E	This study
Rom^R^ Δ*farE* (pCX**-***farR*)	*far*R	This study
***Escherichia coli***		
DC 10B		Monk and Foster, 2012
**Plasmids**		
pBASE6		Geiger et al., 2012
pBASE-*farE*		This study
pCX-30		Strauss and Götz, 1996
pCX-*farR*		This study

### Isolation of a Rom^R^ Mutant in *S. aureus* HG001 and Sequencing Its Genome

For the isolation of a spontaneous Rom-resistant mutant we cultivated *S. aureus* HG001 aerobically in TSB medium for 4 h to active the strain. This culture was used to inoculate (10%) fresh TSB supplemented with 2 μg/ml Rom (4 MIC) and continued the cultivation for 72 h. Then, the culture were subsequently cultured with 10% inoculation into fresh TSB supplemented with 2 μg/ml Rom (4 MIC) and continued the cultivation for 24 h. When plated on TS-agar containing increasing concentrations of Rom, colonies were obtained that were resistant to >128 μg/ml Rom. The resistant clones (Rom^R^) were stable, and retained their resistance even after up to 10 passages in TSB without Rom.

### Comparative Genome Sequencing

Isolation of the chromosomal DNA from the parent strain HG001 and its Rom^R^ mutant and sequencing of both genomes was performed by the DSMZ, Braunschweig (Germany). The long-read sequencing technique PacBio RS II with an average read length of 10 kb was used (Rhoads and Au, 2015). For error correction Illumina sequencing was performed. Aligning both genomes in MAUVE showed one single nucleotide polymorphism (SNP) in CDS called SAUOHSC_02867.

### RNA Isolation for RNA-Seq

*Staphylococcus aureus* HG001 wild type and the Rom^R^ clones were cultured in basic medium for 4 and 8 h. The bacterial cell cultures were harvested by centrifugation at 4,500 × *g* and 4°C for 10 min. The cell pellet was resuspended in 1 ml of acid guanidinium thiocyanate-phenol-chloroform solution (Trizol) and transferred to 2 ml screw cap containing 0.5 ml of 1 mm silica beads. The cells were lysed 3 times in fastprep at 6.5 m/s for 30 s. The samples were cooled on ice for 5 min after each run. The lysate was incubated for 5 min at RT and then 200 μl of chloroform was added. The sample was vigorously shaken for 30 s to extract RNA, incubated at RT for 3 min then centrifuged for 15 min at 15,000 × *g* and 4°C. 500 μl of RNase free isopropanol was added and the aqueous phase was transferred into fresh RNase-free 1.5 ml reaction tubes. The RNA was precipitated by inverting several times and incubating at RT for 10 min. The sample was centrifuged at 15,000 × *g*, 4°C for 15 min and the supernatant was removed by pipetting. 1 ml of 70% RNase free ethanol was added, centrifuged at 7,500 × *g*, 4°C for 5 min and supernatant was discarded by pipetting. The RNA pellet was air-dried for 30 min and resuspended in 50 μl of RNase free H_2_O. RNA concentration was measured by nanodrop. 70–100 μg of RNA was mixed with 50 μl of 10% DNase I buffer (100 mM Tris, pH 7.5, 25 mM MgCl_2_, 5 mM CaCl_2_) and 10 μl of DNase I (2U/μl). The volume of the mixture was adjusted up to 500 μl with DEPC treated H_2_O and the mixture was incubated for 1 h. After the sample was splitted into two 250 μl in 2 ml reaction tubes, 1 volume (250 μl) of acidic phenol, chloroform and isopentanol (25:24:1) pH 4.5–5 was added and the mixture was vigorously vortexed for 3 min then centrifuged at 15,000 × *g*, 4°C for 30 min. The upper phase was transferred to fresh tube.

1/9 volume (28 μl) of 3 M sodium acetate (pH 5.2) and 2.5–3 volume of pure ethanol (700 μl) were added. The mixture was shortly vortexed and placed at −80°C for 30 min. After centrifugation at 15,000 × *g* and 4°C for 30 min, the supernatant was removed and the pellet was washed by adding 1 ml of 70% ethanol and the sample was centrifuged at 15,000 × *g*, 4°C for 5 min. The supernatant was removed and the pellet was air-dried. The pellet was dissolved in 30 μl DEPC treated H_2_O by vortexing and RNA concentration was measured by nanodrop.

For transcriptomic analysis, batches of 1 μg of total RNA was ribo-depleted with the bacterial Ribo-Zero kit from Illumina. The truseq total RNA stranded kit from Illumina was used for the library preparation. Library quantity was measured by the Qubit and quality was assessed on a Tapestation on a DNA High sensitivity chip (Agilent Technologies). The libraries were pooled at equimolarity and loaded at 2 nM for clustering. Oriented 50 bases single-read sequencing was performed on the Illumina HiSeq 4000 sequencer yielding a minimum of 8 million mapped reads per sample. Final RNA-seq analysis and data analysis were carried out using previous described procedures (Cherkaoui et al., 2017). The raw sequence data were filtered by removing reads containing adapter, reads containing poly-N, and low-quality reads. The filtered reads were aligned against the genome of NCTC8325 (CP000253). The reads mapping was normalized as previously described and expressed as RPKM values (Mortazavi et al., 2008).

### Real-Time qPCR Analysis

Total RNA from HG001, Rom^R^ and Rom^R^ Δ*farE* strains was extracted from 8 h cultures in TSB medium using the RNAeasy Mini Extraction Kit (Qiagen) following the manufacturer’s instructions followed by DNaseI treatment overnight (Promega). The RNA was quantified using NanoDrop 2000 (Thermo Fisher Scientific) and used for qPCR experiments. Expression was detected using the Power Sybr RNA to Ct 1 step kit (Thermo Fisher Scientific) following manufacturer’s instructions with the following oligonucleotides: *psmα*1 5′TCATCGCTGGCATCATTA′3 and 5′CATCGTTTTGTCTCCTG′3. The *gyrB* gene using primers 5′TTAGTGTGGGAAATTGTCGATAAT′3 and 5′AGTCTTGTGACAATGCGTTTACA′3 was used as reference for normalization of expression levels of target genes in each condition. The cycling conditions were as follows: cDNA production 48°C during 30 min, for qPCR denaturation at 95°C for 5 min, 40 cycles at 95°C for 25 s, 60°C for 1 min. Relative changes in the expression of individual genes was obtained using −ΔΔCt method. At least three independent cultures were analyzed for each conditions. RT qPCR was performed using AriaMx3005 (Agilent).

### RNA-Seq Data Accession Number

RNA-seq data have been submitted to ArrayExpress^1^ with the accession number: PRJEB30619.

### Deletion of *farE* Gene by Allelic Replacement in *S. aureus* HG001

**FIGURE 1:**
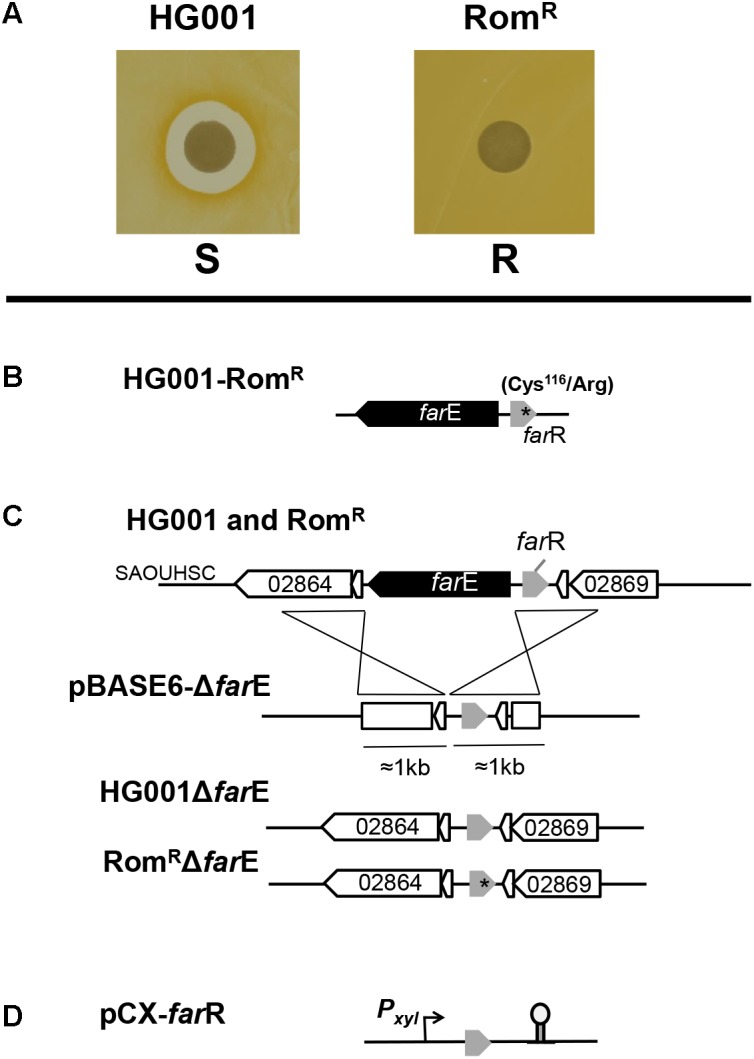
Rom-resistance phenotype, genetic analysis and construction of deletion mutants and clones. **(A)** The Rom^R^ clone shows no inhibition halo in the agar diffusion method with 100 μg Rom loaded on the filter disks. **(B)** Genetic organization of the divers oriented *farR* and *farE* genes and location of the single point mutation (^∗^) in *farR* leading to an amino acid exchange (Cys116/Arg) in the FarR regulator protein in the Rom^R^ clone. **(C)** Illustration of *farE* deletion construction in HG001 and the Rom^R^ clone. For homologous double-cross recombination, pBASE6-Δ*farE* containing approximately 1 kb upstream and downstream DNA sequences were used; Δ*farE* positive clones were controlled by PCR and sequencing. **(D)** pCX30::*farR* is the *farR* complementation plasmid in which the transcription of the gene is xylose-inducible.

The deletion of *farE* gene in *S*. *aureus* HG001 was generated by homologous recombination (Figure 1). The basis for the construction of the knock-out plasmid was temperature-sensitive vector pBASE6, in which two DNA fragments were cloned. Briefly, 1 kb upstream and 1 kb downstream region were amplified by PCR the genome of *S*. *aureus* HG001 using primer pairs F_*farE*up and R_*farE*up for the upstream region and F_*farE*down and R_*farE*down (Supplementary Table S2). Both fragments were purified and ligated into pBASE6 using Gibson assembly (Gibson et al., 2009) according to manufacturer instructions. The ligation reaction was purified and transformed into competent cells of *E. coli* DC10B. The positive plasmids were selected by PCR and sequencing. The selected plasmids were sub-transformed into *S. aureus* HG001 and Rom^R^. Further steps to obtain the marker-less mutant strains were performed as described by Bae and Schneewind (2006). Positive clones were selected and verified by PCR and sequencing.

**Table 2:** Minimal inhibition concentrations (MIC) for Rom.

Strains	MIC (μg/ml)
HG001	0.5
Rom^R^	>128
HG001Δ*farE*	0.5
Rom^R^ Δ*farE*	0.5
HG001 (pCX-*farR*)	0.25
Rom^R^ (pCX-*farR*)	0.25

### Construction of pCX-*farR*

The plasmid pCX30::*farR* (Figure 1D) contains the native *farR* gene (SAUOHSC_0286) under control of the xylose-inducible promoter (Wieland et al., 1995). To construct this plasmid, *farR* gene was amplified by PCR using forward primer and reversed primer F_*farR*(BamHI) and R_*farR*(XmaI) (Supplementary Table S2). In the forward primer, the ribosomal binding site was optimal with the sequence AGGAGGT. The amplified PCR fragment and pCX30 were cut by *Bam*HI and *Xma*I and subsequently ligated. The ligation product was first transformed into *S. aureus* RN4220. The positive pCX-*farR* plasmid was subsequently transformed into the Rom^R^ clone for further analysis.

### Determination of MIC

Minimal inhibitory concentration values of Rom were determined by a modified broth microdilution method in 96-well microtiter plates using Müller-Hinton broth in a final volume of 200 μl. The inoculum size, 10^5^ cells ml^−1^ were used as described by Saising et al. (2018). The concentration gradient of Rom ranged from 128 to 0.125 μg/ml. 100 μ of bacterial suspension was inoculated and incubated at 37°C for 16–18 h. The MIC was read at the lowest concentration that completely inhibited the bacterial growth. Xylose (0.5%) was added to the medium for *S. aureus* HG001(pCX-*far*R) and Rom^R^(pCX-*far*R). Rom resistance also was determined by Kirby–Bauer antibiotic testing. 100 μl of 10^8^ CFU/ml of bacteria were spread on the TSA plates. 100 μg of Rom was loaded on the paper filters. Pictures were taken after 18 h incubation at 37°C. Inhibition zones surrounding the filter disks indicate susceptibility to Rom (Figure 1A and Table 2).

### Analysis of Release of Lipids

Release of lipids into the bacterial supernatant was carried out as described by Pader et al. (2016). Briefly, lipids were detected and quantified using FM5-95 (Thermo Fischer). The bacterial strains were cultivated in TSB medium for 8 or 16 h. The OD was determined and cultures were equally adjusted to the same OD. Then equal aliquots of cells were centrifuged and the supernatants filtered. 100 μl of each sample were mixed with FM5-95 to a final concentration of 5 μg/ml. Fluorescence was measured with a Tecan microplate reader using excitation at 565 nm and emission at 660 nm.

### Fatty Acid and Lipid Extraction and Analysis by GC-MS

Bacterial strains were cultivated in BM medium for 16 h. The cultures were adjusted to the same OD, the cells were centrifuged and the supernatant was used for FA determination. The total lipids and free FAs were extracted from filtered supernatant by the Bligh and Dyer method (Bligh and Dyer, 1959). Samples were prepared for GC-MS analysis. In detail, dried supernatant extract was suspended in 1 ml reagent I (22.5 g NaOH + 75 ml MeOH + 75 ml water) and transferred to 10 ml glass jars with screw top lids with a Teflon seal for saponification. The suspension was well vortexed and incubated for 35 min at 100°C. After cooling, 2 ml of reagent II (162.5 ml 6 N HCl + 137.5 ml MeOH) was added for esterification. The suspension was incubated for 12 min at 80°C and cooled down again. The FA methyl esters were extracted by adding 1.25 ml of Hexan. The suspension was then shaken for 5 min, until phase separation occurred. After a short centrifugation step, the upper phase was transferred to a fresh glass vial and used for GC-MS. Before FA analysis, 0.2 mg/ml of C12:0 in ethanol (99%) was used for GC as an internal standard [C12 FAs were not found in *S. aureus* (Nguyen et al., 2015)]. GC analysis was carried out by Gas Chromatograph (GC) (Hewlett Packard HP 6890) with a DBWAX-30 W column [covalent bond phase, length: 30 m, i.d. 0.139 mm (Macherey-Nagel, Düren)]. Temperature program was 3 min isotherm 80°C, 80–250°C at 10°C min^−1^ and 5 min at 250°C. GC parameters were: injector temperature, 250°C; detector temperature, 250°C; column pressure, 450 kPa, injector pressure, 100 kPa; flame ionization detector, 60 kPa; air, 30 kPa; slpit, 1:19.2.

### Analysis of the Excreted Cytoplasmic Proteins

FbaA, aldolase; GAPDH, Glyceraldehyde-3-phosphate dehydrogenase are typical cytoplasmic proteins, which can be excreted into the supernatant (Ebner et al., 2015). For detection of FbaA and GAPDH activity in the supernatant. 16 h grown cultures were diluted to the same OD (OD_578_ = 10). Then, 2 ml was sterile filtered (0.45 μm pore size) and 50 μl were used for the assay. For the detection, Aldolase Activity Assay Kit (Colorimetric) and Glyceraldehyde-3-Phosphate Dehydrogenase Activity Assay Kit (Colorimetric) (both from Abcam) were used. Both assays were done following the manufacturer description.

### Impact of Bacterial Supernatant on Cytotoxicity

The cytotoxic capacity of the supernatant was determined in three well characterized human cell lines: HaCaT, a spontaneously transformed aneuploidy immortal human keratinocyte cell line, HEK 293, human embryonic kidney cells, and A549, human lung carcinoma cells. The three cell lines were cultured in DMEM-high glucose medium (Life Technology, Darmstadt) supplemented with 10% FBS (Biochrom, Berlin) and 1% Pen/Strep (10 mg/ml) at 37°C with 5% CO2. Cytotoxicity was determined by using the LDH cytotoxicity assay kit (Thermo Scientific). All experiments were performed triplicate in a 96 well assay plate. Bacteria were grown for 16 h in TSB medium and were subsequently diluted to the same OD_578nm_ 10. The cells were centrifuged and the supernatant was filtered (0.45 μm pore size). For LDH cytotoxicity assay, 100 μl of supernatant was added to the human cells and incubated for 3 h at 37°C with 5% CO_2_. As negative control served wells containing only DMEM/F; and for positive controls 10 μl lysis buffer was added. After incubation the cell suspensions were centrifuged and the supernatant was transferred to a new 96-well flat bottom plate, subsequently mixed gently with reaction mixture and incubated at room temperature in the dark for 30 min. Finally, the stop solution was added and shortly centrifuged to break any bubbles. The samples were measured at a wavelength 490 and 680 nm by Tecan Infinite 200 (Tecan, Männedorf CH) plate reader.

### Mouse Infection Experiments

Overnight cultures of HG001 and Rom^R^ in BHI medium were diluted to a final OD_600_ of 0.05 in 50 mL fresh BHI medium and grown for 3.5 h at 37°C. After centrifugation, the cell pellet was resuspended in BHI with 20% glycerol, aliquoted and stored at −80°C. For the generation of *in vivo* infection, aliquots were thawed and washed twice with PBS. The infectious dose used for infection was very similar for both strains (4 × 10^8^ CFU/HG001 and 3.5 × 10 CFU/Rom^R^ in 20 μl). A sample of the infection inoculum was plated on TSB agar plates in order to control the infection dose. For the intranasal *S. aureus* infection model (pneumonia model) we used female Balb/c mice (9 per group, 6 weeks, Janvier Labs, Le Genest-Saint-Isle, France). They were intranasally infected with 4 × 10^8^ CFU of HG001 and with 3.5 × 10^8^ CFU of Rom^R^. During infection, mice were scored twice a day and the severity of infection was determined accordingly. After 48 h of infection, mice were sacrificed, the lungs recovered, homogenized and plated in serial dilutions on TSB agar plates in order to determine the bacterial burden. Significant difference in the CFU counts in the lungs between both two groups was determined with Mann–Whitney-test (software: GraphPad Prism 5.0): two-tailed: 0.0625 = n.s.; one-tailed: 0.0313 = ^∗^.

### Ethics Statement

All of the animal studies were approved by the local government of Franconia, Germany (approval number 55.2-2532-2-155) and performed in strict accordance with the guidelines for animal care and experimentation of German Animal Protection Law and the DIRECTIVE 2010/63/EU of the EU. The mice were housed in individually ventilated cages under normal diet in groups of four to five throughout the experiment with *ad libitum* access to food and water.

### Statistical Analysis

Analysis of variance (ANOVA) test or Student’s *t*-test and Mann–Whitney test were employed when appropriate to compare the difference of means between the mutant clones with the wild-type HG001 when it appropriates. All the statistical analysis was performed by GraphPad Prism. The significance level was set as follows: a *P*-value of >0.05 was considered not significant (ns). In figures, significant differences are depicted as follows: ^∗^*P* < 0.05, ^∗∗^*P* < 0.01, ^∗∗∗^*P* < 0.001, ^∗∗∗∗^*P* < 0.0001.

## Results

### Isolation of a Rom Resistant Mutant (Rom^R^) From *S. aureus* HG001

To learn more about the mechanism of Rom’s interaction with *S. aureus* we tried to isolate a Rom^R^ mutant *S. aureus* HG001. For this we streaked ≈ 5 × 10^9^ cells of an HG001 overnight culture on TS agar plates containing 5, 10, and 20 μg/ml Rom. However, no Rom^R^ colonies arose. Only after preceding several subcultivations of HG001 in TSB medium containing 2 μg/ml Rom (4xMIC) spontaneous Rom^R^ mutants could be reproducibly isolated. The Rom^R^ clones were completely devoid of an inhibition zone on the agar diffusion assay (Figure 1A). Indeed, the Rom^R^ clones were highly resistant to Rom, showing MIC values > 128 μg/ml (Table 1).

### A Single Point Mutation in the *farR* Gene Caused Rom^R^

By comparative whole genome sequencing of HG001 and its Rom^R^ it turned out that there was only one single point mutation in the genome of Rom^R^. In the ORF SAUOHSC_02867 the cysteine codon TGC116 of the HG001 was mutated to the arginine codon CGC in Rom^R^ (Figure 1B). Besides the Cys116/Arg mutant we obtained in two further independent screenings spontaneous Rom-resistant mutants with amino acid exchanges in Val115/Ile and Phe153/Leu, respectively. All the mutants were stable over more than 10 passages. The ORF SAUOHSC_02867 represents the *farR* gene, which encodes a regulator with an N-terminal TetR family DNA binding domain (Alnaseri et al., 2015). FarR controls the expression of the divergently transcribed *farE* (effector of fatty acid resistance). FarE, a membrane protein with 12 predicted transmembrane domains (Supplementary Figure S2), is described to represent a multidrug efflux pump (Alnaseri et al., 2015).

Detailed information on the expression of the *farR* and *farE* genes was obtained in a study analyzing the transcriptome of *S. aureus* HG001 under more than 40 different experimental conditions, including different growth stages in complex and minimal media as well as infection-related conditions like growth in human serum, oxygen limitation or internalization by eukaryotic cells (Mäder et al., 2016). Inspection of *farR* and *farE* transcript levels under these conditions revealed that *farR* is significantly and evenly expressed under all conditions tested (Supplementary Figure S3). In contrast, expression of *farE* is only observed during exponential growth of *S. aureus* in various cultivation media (TSB, RPMI, and MEM), whereas during growth in chemically defined medium or human plasma, in stationary phase cells or after internalization into host cells expression levels are very low.

To confirm that the mutation in *farR*^∗^ is responsible for the Rom^R^ phenotype and to investigate the contribution of *farE* we constructed various deletion mutants and plasmids (Figure 1C). To see whether *farR* or the expression of *farE* by its proposed regulator FarR was responsible for Rom^R^, we deleted *farE* in both HG001 and Rom^R^ strains. If *farE* is deleted in Rom^R^Δ*farE*, the clone became as sensitive to Rom as the parent HG001 (Table 2), suggesting, that FarE is ultimately responsible for the Rom^R^ phenotype. Deletion of *farE* in the parent strain, HG001Δ*farE*, showed no effect. We also cloned the parent (none-mutated) *farR* in a xylose inducible plasmid, pCX-*farR* (Figure 1D), and transformed the plasmid into HG001(pCX-*farR*) and Rom^R^(pCX-*farR*). As shown in Table 2 both clones became hypersensitive to Rom, suggesting that FarR is acting as a repressor of *farE* expression, and that the point mutation in *farR*^∗^ inactivates the repressor function.

### Comparative Transcriptome Analysis Disclosed Up- and Down-Regulated Genes in Rom^R^ Clone (*farR* Mutant)

**FIGURE 2:**
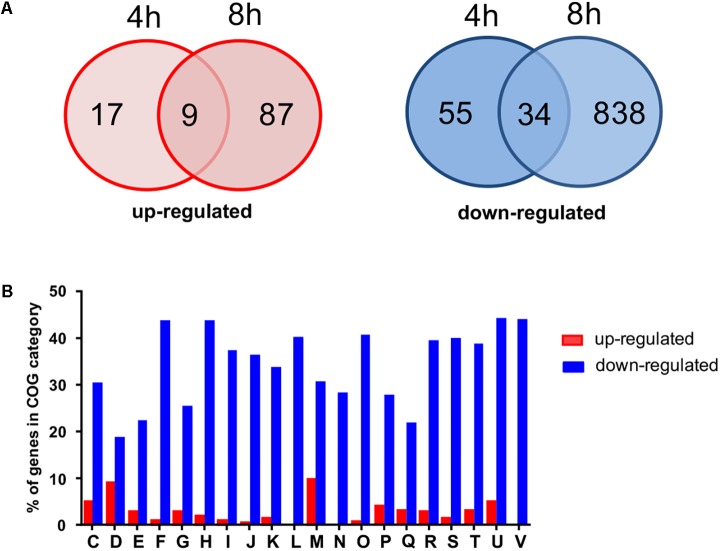
Transcriptome analysis (RNA-seq) of genes differently expressed in Rom^R^ clone compared to HG001 wild type strain. **(A)** The Venn diagrams show numbers of genes that are at least fourfold up- or downregulated after 4 and 8 h of growth in BM in the Rom^R^ clone. **(B)** Percentage of genes being at least fourfold up (red bars) or downregulated (blue bars) in each of cluster orthologous groups (COG) based on functional categories. Designations of functional categories: C, energy production and conversion; D, cell division and chromosome partitioning; E, amino acid metabolism and transport; F, nucleotide metabolism and transport; G, carbohydrate metabolism and transport; H, coenzyme metabolism; I, lipid metabolism; J, translation, ribosomal structure and biogenesis; K, transcription; L, DNA replication, recombination, and repair; M, cell wall structure and biogenesis, outer membrane; N, Cell motility and chemotaxis; O, post-translational modification, protein turnover, chaperone functions; P, Inorganic ion transport and metabolism; Q, secondary metabolites biosynthesis, transport and catabolism; R, general functional prediction; T, signal transduction mechanisms; U, secretion; V, defense mechanisms.

For the initial identification of potential cellular processes affected by gene expression alterations in the Rom^R^ clone, we compared the transcriptomes of HG001 and Rom^R^ clones after 4 h (early log phase) and 8 h (early stationary phase) of growth. The most pronounced differences were seen after 8 h. In total, approximately 1000 genes were ≥4-fold differentially expressed in the Rom^R^ clone with the point mutation in *farR*^∗^ compared to the parental strain. More than 100 genes were up- and 900 genes were down-regulated in Rom^R^ (Figure 2A). The total list of gene expression data is given in Supplementary Table S1. The genes that are differently expressed can be directly or indirectly (by other regulators) controlled by FarR. We categorized the genes according to their functional classification into 20 clusters of orthologs (COG) categories (Tatusov et al., 2003) (Figure 2B). The results indicate that FarR is an important regulator. Some of the most up- or down-regulated genes in the *farR* mutant (Rom^R^) are listed in Table 3. We indicated the ratio Rom^R^ versus WT. Genes that are higher expressed in the *farR* mutant (Rom^R^) (ratios > 1) are negatively controlled by FarR, while genes that are lower expressed are positively controlled by FarR.

**Table 3:** Selected annotated genes that are up- or down-regulated in Rom^R^ clone^1^.

Gene^2^	Function	Ratio Rom^R^/WT	
		4 h	8 h
**Regulators**			
SAOUHSC_02867	FarR-regulator protein (*farR*)	5.40	18.27
SAOUHSC_02261	Accessory gene regulator protein B (*agrB*)	1.51	5.83
SAOUHSC_02260	Delta hemolysin (*hld*), RNAIII	17.66	n.d.
SAOUHSC_00620	Accessory regulator A (*sarA*)	1.56	3.77
SAOUHSC_00666	Glycopeptide resistance-associated two-component system, GraS (*graS*) and	0.61	0.08
SAOUHSC_00665	GraR (*graR*)	0.49	0.11
**Secreted toxins, enzymes and transporters**			
SAOUHSC_01121	Alpha-hemolysin (*hla*)	0.7	97.5
SAOUHSC_01135	Phenol-soluble modulin beta1 (*psm*ß1)	231.1	1113.5
SAOUHSC_03006	Lipase (SAL1) (*gehA*)	0.33	127.7
SAOUHSC_00987	Cysteine protease, staphopain B (*sspB*)	1.44	7.30
SAOUHSC_02866	FarE, fatty acid efflux pump (*farE*)	19.46	92.82
SAOUHSC_02874	Cation transporter E1-E2 family ATPase	4.25	5.75
SAOUHSC_01193	Fatty acid kinase (*fakA*)	0.46	0.15
SAOUHSC_00764	FA-binding protein, binds saturated FA (*fakB1*)	0.89	0.07
SAOUHSC_01433	FA-binding protein, prefers unsaturated FA (*fakB2*)	0.28	4.38
**Capsular proteins**			
SAOUHSC_00114	Capsular polysaccharide biosynthesis protein	0.94	32.25
SAOUHSC_00115	Capsular polysaccharide biosynthesis Cap5B	1.47	11.82
SAOUHSC_00116	Capsular polysaccharide biosynthesis Cap8C	2.99	12.46
**Cell wall bound proteins**			
SAOUHSC_00069	Protein A (*spa*)	1.03	0.08
SAOUHSC_01175	Fibrinogen-binding protein A-like protein (*sdrA*)	0.62	0.15
SAOUHSC_00544	Fibrinogen-binding protein SdrC (*sdrC*)	0.16	0.09
SAOUHSC_00545	Fibrinogen-binding protein SdrD (*sdrD*)	0.37	0.09
SAOUHSC_02855	LysM domain-containing protein	0.20	0.01

*^1^Transcriptome data analysis is based on the NCBI RefSeq annotation of Staphylococcus aureus NCTC 8325, the progenitor strain of HG001. ^2^Total genes are listed in Supplementary Table S1. Gene names were obtained from AureoWiki (Fuchs et al., 2018). n.d, not determined.*

**FIGURE 3:**
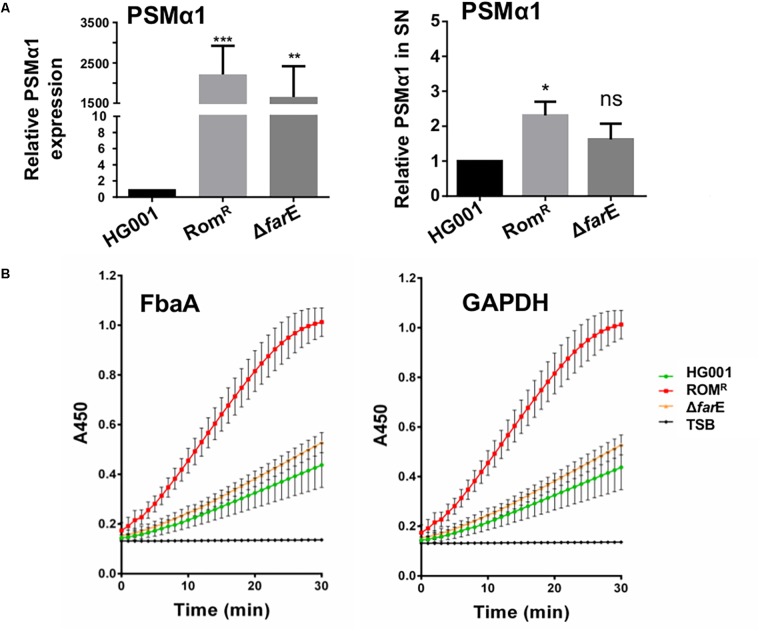
Comparison of release of PSMα and the cytoplasmic proteins FbaA and GAPDH. **(A) (Right)** PSMα1 expression of HG001, Rom^R^ and Δ*farE* (Rom^R^Δ*farE*) were determined by Real-Time PCR. The strains were aerobically cultured in TSB medium for 16 h. The pellets were used for total RNA extraction. qPCR experiments were carried out to determinate *psmα*1 and *gyrB* was used as housekeeping gene. Fold changes were calculated using ΔΔCt method and relativized to the HG001 expression. Error bars indicate standard error. Statistical significances between mutant clones Rom^R^, Δ*farE* (Rom^R^ Δ*farE*) with the wild type HG001 were analyzed by 1-way ANOVA: not significant *P* > 0.05, ^∗^*P* < 0.05, ^∗∗^*P* < 0.01, ^∗∗∗^*P* < 0.001. **(Left)** Release of PSMα1 into the supernatant of HG001, Rom^R^ clone and Rom^R^Δ*farE* cultured in TSB for 16 h was determined by HPLC; the relative amount of PSMα was calculated by comparing the peak-area in the samples. All experiments were performed in triplicate. Error bars indicate standard error. Statistical significances between mutant clones Rom^R^, Δ*farE* (Rom^R^ Δ*farE*) with the wild type HG001 were analyzed by Student *t*-test: not significant *P* > 0.05, ^∗^*P* < 0.05, ^∗∗^*P* < 0.01, ^∗∗∗^*P* < 0.001. **(B)** Comparative release of FbaA (aldolase) and GAPDH (glyceraldehyde-3-phosphate dehydrogenase) over time in HG001, Rom^R^ clone and Rom^R^Δ*farE* clone. Only TSB was used as control.

Comparing the effect of FarR on other global regulators it turns out that it negatively controls its own expression (*farR*), the accessory gene regulator (*agr*) operon, and the accessory regulator A (*sarA*); while the glycopeptide resistance-associated two-component system (*graSR*), which was lower expressed in the *farR* mutant (Rom^R^), appears to be positively controlled by FarR (Table 3).

In the Rom^R^ clone genes were upregulated that encode toxins (*hla* and *psmβ1*), secreted enzymes (*gehA* and *sspB*), membrane bound transporters (*farE* and a cation transporter E1–E2) or capsular polysaccharide biosynthesis genes. That means that all these genes are negatively regulated by FarR. As the *psm-α* genes are not annotated in the HG001 genome (NCTC 8325 background) we compared the mRNA expression of *psm-α1* by qRT-PCR analysis and could demonstrate that the PSMα expression is about 2,000 times higher of the Rom^R^ than that of the parent strain HG001 (Figure 3A). In contrast to the mentioned secreted proteins several cell wall bound proteins such *spa*, *sdrACD* and a LysM domain-containing protein gene were lower expressed in the *farR* mutant (Table 3). The upregulation of secreted proteins and the down-regulation of cell wall anchored proteins in the Rom^R^ clone (*farR* mutant) suggest that FarR negatively regulates the global regulators *agr* and *sarA* (Wolz et al., 2000; Novick, 2003).

### In the Rom^R^ Clone (*farR* Mutant) Secretion of Virulence Factors and Release of Cytoplasmic Proteins Is Increased

According to the transcriptome analysis, a number of virulence genes were upregulated in Rom^R^ clone (Table 3). Among them were lipase (*geh*), alpha-hemolysin (*hla*), and phenol soluble modulins (*psm*) genes. We can show that the increased transcription was also correlated with increased expression of the corresponding proteins. For example, the hemolysis halo on blood agar was larger in Rom^R^ compared to HG001 (Supplementary Figure S1A) and in the lipase zymogram the lipase activity bands were more pronounced in Rom^R^ compared to HG001 (Supplementary Figure S1B) or Protein A was expressed lower in ROM^R^ than that in HG001 (Supplementary Figure S1C). Regarding the phenol soluble modulins (PSMs) particularly the α-types are highly cytotoxic (Peschel and Otto, 2013). Here we show that the amount of PSMα1 was about 2.5-fold higher in the Rom^R^ clone compared to the parent HG001 (Figure 3A).

As it is known that the expression of PSMα peptides significantly increased the excretion of cytoplasmic proteins (ECP) (Ebner et al., 2017), we investigated whether the prototype cytoplasmic proteins, Fba and GAPDH, were also more abundant in the supernatant of Rom^R^. The amount of both enzymes was indirectly determined by assaying their activity. Indeed, the activity of both enzymes was much higher in Rom^R^ than in HG001 or Rom^R^Δ*farE* (Figure 3B). These results corroborate the correlation between PSMα production and ECP.

### Derepression of *farE* in the Rom^R^ Clone Increased Release of Lipids and FAs

**FIGURE 4:**
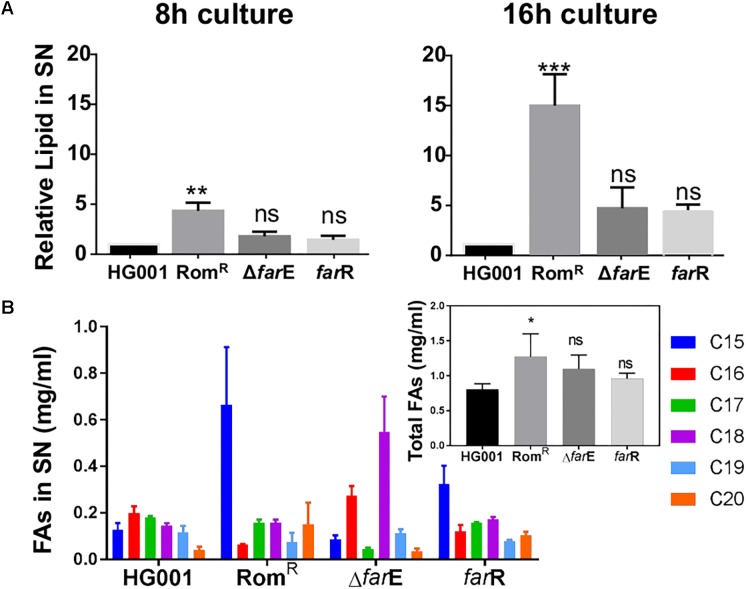
Comparison of release of lipids and total FAs into the supernatant. **(A)** Release of lipids. The *S. aureus* clones (Rom^R^ clone, Rom^R^Δ*farE*, and Rom^R^Δ*farR*) were aerobically cultured in TSB medium for 8 and 16 h. The supernatants were filtrated and 100 μl of each sample were mixed with FM5-95 to a final concentration of 5 μg/ml. Fluorescence was measured with a Tecan microplate reader using excitation at 565 nm and emission at 660 nm. All experiments were performed in triplicate. The relative lipids in the supernatant were relativized to the HG001 value. Error bars indicate standard error. Statistical significances between mutant clones Rom^R^, Δ*farE* (Rom^R^ Δ*farE*), *farR* [Rom^R^ (pCX-*farR*)] with the wild type HG001 were analyzed by 1-way ANOVA: not significant *P* > 0.05, ^∗^*P* < 0.05, ^∗∗^*P* < 0.01, ^∗∗∗^*P* < 0.001. **(B)** Comparison of FAMEs (C15 to C20). The FAs in the supernatants of 16 h cultures of HG001, Rom^R^ clone, ΔfarE (Rom^R^Δ*farE*), and farR [Rom^R^ (pCX-*farR*)] were qualitatively and quantitatively analyzed by GC-MS. The FAs from C15 to C20 were assigned different colors. The total FAs for each clone are shown in the inserted graph. Experiments were performed independently in duplicate. Error bars indicate standard error. Statistical significances between mutant clones Rom^R^, Δ*farE* (Rom^R^ Δ*farE*), *farR* [Rom^R^ (pCX-*farR*)] with the wild type HG001 were analyzed by 1-way ANOVA: not significant *P* > 0.05, ^∗^*P* < 0.05, ^∗∗^*P* < 0.01, ^∗∗∗^*P* < 0.001.

As FarE is described as an efflux pump for linoleic and arachidonic acids (Alnaseri et al., 2015), we determined the release of lipids into the supernatant in various clones by using the fluorescent dye FM5-95 that preferentially binds to bacterial lipids. Cells were cultivated in TSB and the supernatant was taken from 8 h- and 16 h cultures. We compared the release of lipids in HG001, Rom^R^, Rom^R^Δ*farE*, and Rom^R^(pCX-*farR*); in the latter native *farR* is xylose-inducible expressed on a plasmid (Figure 1). At both time points the Rom^R^ clone exhibited the highest amount of released lipids in the supernatant (Figure 4A). In the 16 h culture sample the lipid content was 15× higher than in HG001. If we delete *farE*, or, if we overexpress native *farR* in Rom^R^ clone the amount of released lipids was decreased significantly, close to the level of the parent strain HG001 (Figure 4A). These results confirm that the derepression of *farE* plays a crucial role in Rom resistance, which is supported by the fact that overexpression of FarE enhanced excretion of lipids.

We also investigated the fatty acid methyl ester (FAME) hydrolyzed from the total released lipids by GC-MS analysis in various clones. Interestingly, in the RomR clone two FAs, namely C15 (pentadecylic acid) and C20 (arachidic acid), were increased 5- and 3.7-fold compared to HG001, and 7.6- and 4.3-times higher than in Rom^R^Δ*farE* (Figure 4B). On the other hand the content of C16 (palmitic acid) was lower in the Rom^R^ clone by about threefold compared to HG001 and the Rom^R^Δ*farE* mutant. The total amounts of FAME in the supernatants were significantly higher in the RomR clone (Figure 4B, inserted graph). The results indicate that released lipids are qualitatively and quantitatively altered in the Rom^R^ clone compared to the other clones.

### The Supernatant of Rom^R^ Clone Was More Cytotoxic Than That of the Parent Strain HG001

**FIGURE 5:**
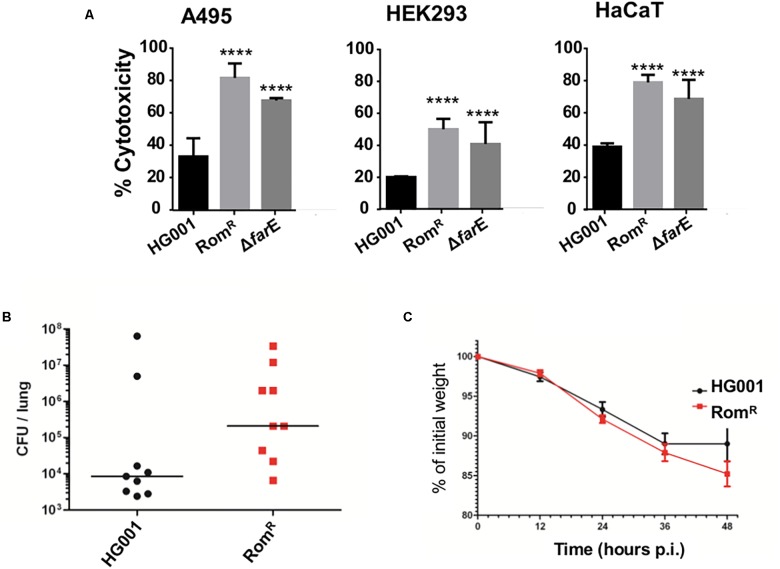
Comparison of cytotoxicity and mouse pathogenicity. **(A)** Comparison of the cytotoxicity of HG001, Rom^R^ clone, and Rom^R^Δ*farE* mutant in various host cells A495, HEK, and HaCaT. Experiments were performed in triplicate. Error bars indicate standard error. Statistical significances between mutant clones Rom^R^, Δ*farE* (Rom^R^ Δ*farE*) with the wild type HG001 were analyzed by 1-way ANOVA: not significant *P* > 0.05, ^∗^*P* < 0.05, ^∗∗^*P* < 0.01, ^∗∗∗^*P* < 0.001, ^∗∗∗∗^*P* < 0.0001. **(B,C)** Comparison of HG001 and Rom^R^ clone in 48 h intranasal mouse infection model (pneumonia model). The Rom^R^ clone shows higher CFU values in the lungs after 48 h of infection **(B)**, and a slightly stronger weight loss than the wild type HG001 **(C)**. Significant differences (^∗^*P* = 0.0313) in bacterial burden were noted between Rom^R^ and the wt HG001. Data were analyzed using Mann–Whitney one tailed test.

Since the Rom^R^ clone produced more toxins we investigated the cytotoxic activity in the supernatants of HG001, Rom^R^ and Rom^R^Δ*farE* grown for 16 h in TSB. Cytotoxicity was determined in three human cell lines: HaCaT, HEK 293, and A549 by assaying the release of cytoplasmic lactate dehydrogenase (LDH) from lysed cells. As shown in Figure 5A the supernatant of Rom^R^ showed in all cell lines a significant higher cytotoxicity than its parent HG001. In the Rom^R^Δ*farE* clone the cytotoxicity was slightly decreased compared to Rom^R^ (Figure 5A).

### In the Mouse Infection Model (Pneumonia) the Rom^R^ Clone Was More Pathogenic Than the Parent HG001

Finally, we investigated whether the Rom^R^ clone is also more pathogenic *in vivo*. For the *S. aureus* infection model (pneumonia model) female Balb/c mice (9 per group, 6 weeks, Janvier Labs, Le Genest-Saint-Isle, France) were used. They were intranasally infected with 4 × 10^8^ CFU of HG001 and with 3.5 × 10^8^ CFU of the Rom^R^ clone. Indeed, the Rom^R^ clone showed a higher CFU load in the lungs after 48 h of infection (Figure 5B), and slightly stronger weight loss than the parent HG001 (Figure 5C). This result indicates that the Rom^R^ clone was more pathogenic than its parent strain HG001.

## Discussion

We isolated a Rom-resistant mutant in *S. aureus* cultures performed in the presence of sub-lethal concentration of the drug, which differed from its parent only from one point mutation in the *farR* gene causing an amino acid exchange (Cys116Arg) in FarR. *farR* (regulator of FA resistance) and the neighboring *farE* (effector of FA resistance) have been recently described by Alnaseri et al. (2015). FarE causes resistance to the antimicrobial FAs arachidonic and linoleic acids by promoting their efflux. FarR acts as a repressor of *farE* and, consequently, when *farR* was mutated *farE* was up-regulated thus increasing the resistance to FAs. The FarR/FarE system is similar to the *E. coli acrR*/*acrB* counterpart involved in resistance to acriflavine and ciprofloxacin (Nakamura, 1968; Ma et al., 1995; Webber et al., 2005).

A similar mechanism is underlying the high resistance to Rom as the described resistance to the FAs in *S. aureus*. The point mutation in *farR* in *S. aureus* inactivates the repressor function of FarR with the effect that *farE* becomes de-repressed and more FarE can be produced. Our transcriptome analysis shows that FarR is a strong repressor of *farE*. We assume that the derepression of *farE* is causative for the high Rom resistance. This assumption is corroborated by two results: (a) deletion of *farE* in the Rom^R^ clone (Rom^R^ Δ*farE*) makes the clone Rom-sensitive like the parent strain, and (b) cloning of native *farR* in the Rom^R^ clone (Rom^R^ (pCX-*farR*)) makes the clone hyper-sensitive to Rom.

All data speak in favor that overexpression of FarE is responsible for the high Rom resistance; however, the underlying mechanism is not fully clarified. Currently, we are considering two possibilities by which Rom resistance is mediated by FarE: (a) FarE excretes FA/lipids that antagonize or neutralize certain antibiotics like Rom, or (b) FarE is an efflux pump for certain antibiotics like Rom. In both cases antibiotic susceptibility would be altered.

In favor for assumption (a) speaks that it has been shown that the addition of saturated FAs (nC15:0, nC16:0, and nC18:0) can partially counteract Rom’s antimicrobial activity (Saising et al., 2018). Another evidence for the first hypothesis is that the Rom^R^ clone excretes significantly more lipids and total FAs into culture supernatant than the parent strain HG001 or the Δ*far*E (Rom^R^ Δ*farE*) and *far*R (Rom^R^ pCX-*far*R) clones. Although FarE appears to play a major role in Rom resistance there might be also other factors that play a role. For example in the Rom^R^ clone the excretion of PSMα1 and also of cytoplasmic proteins such as FbaA and GAPDH is increased suggesting that the integrity of the cytoplasmic membrane is disturbed. Particularly the cytotoxic PSMα peptides can mobilize lipoproteins, the TLR2 agonists, from the staphylococcal cytoplasmic membrane and boost excretion of cytoplasmic proteins (Hanzelmann et al., 2016; Ebner et al., 2017). The enhanced expression of agr in the Rom^R^ clone indicates that FarR is a repressor of agr and also of sarA. Therefore, we assume that one of the most important functions of FarR is the balancing of the expression of global regulators such as agr and SarA; by this FarR becomes itself, indirectly, a global regulator. We don’t know yet whether lipids, FAs or other released compounds in Rom^R^ clone cause Rom resistance, although, certain lipids and FAs are hot candidates.

**FIGURE 6:**
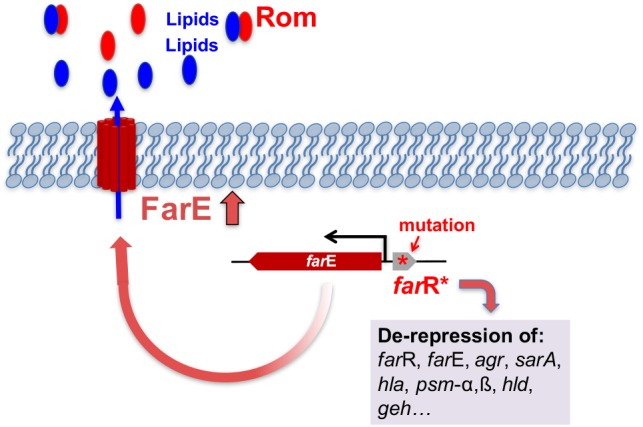
Model for FarE mediated resistance to Rom in the Rom^R^ clone. In the Rom^R^ clone *farR* is inactivated by the point mutation that leads to an amino acid exchange (Cys116/Arg) in the FarR regulator protein. FarR, acts not only as a repressor of its own *farR* gene but also represses *farE*. The mutation in *farR* indicated by (^∗^) leads to inactivation of FarR and consequently to derepression of *farE* and many other genes (including *farR*, *agr*, *sarA*, *hla*, *psm*-*α*,*β*, *hld*, *geh*). Derepression of *farE* causes over-expression of FarE. We propose that FarE acts as an exporter of lipids that interact with Rom thus causing neutralization of Rom and leading to high Rom resistance.

We assume that the increased excretion of lipids neutralize Rom’s activity which would be supported by the previous finding that Rom binds transiently to phospholipid head groups (Saeloh et al., 2018). We furthermore assume that the excreted lipids come from membrane turnover metabolism. In agreement with this assumption is that *S. aureus* releases phosphatidylglycerol (PG) during the stationary phase of growth (Short and White, 1971), and keeps an intracellular non-esterified FA pool that is elevated in strains lacking FA kinase activity (Ericson et al., 2017). FA kinase (Fak) is ubiquitous in Gram-positive bacteria consisting of an ATP-binding protein (FakA) that phosphorylates the FA bound to FakB (FakB1 or FakB2) (Parsons et al., 2014). When comparing the expression profile of *fakA* and *fakB2* genes in the Rom^R^ clone and the wild type, it turned out that in the Rom^R^ clone both genes are repressed, suggesting that the intracellular non-esterified FA pool in the Rom^R^ clone might be also increased. It is therefore well possible that part of the non-esterified FA is exported by FarE and thus contributing to neutralization of Rom.

The cytotoxicity of the Rom^R^ clone was significantly higher than that of the parent strain HG001 or the Δ*farE* mutant. In the Rom^R^Δ*farE* mutant the difference was less pronounced suggesting that it is essentially the point mutation in the *farR* gene that makes the Rom^R^ clone less cytotoxic. FarR belongs to the TetR family of transcriptional regulators and shows similarity to AcrR of *E. coli* (Ramos et al., 2005; Alnaseri et al., 2015). The point mutation in *farR* led to an exchange of cystein 116 by arginine. As the regions around Cys^116^ in FarR and Cys^148^ in AcrR of *E. coli* are highly conserved, we assume that the Cys^116^ is important and that the amino acid exchange in FarR most likely causes its inactivation.

Our comparative transcriptome analysis (HG001 against its Rom^R^ clone) reveals that FarR controls directly or indirectly roughly 1000 genes that were ≥4-fold differentially expressed. No question, that FarR is a regulator, which affects positively and negatively the expression of genes involved in various cellular processes.

However, the important point for the understanding of the increased cytotoxicity of the Rom^R^ clone is the derepression of various other regulators that are repressed by an intact FarR. Interestingly, in the Rom^R^ clone the *farR* gene is derepressed, indicating that FarR negatively regulates its own expression. Furthermore, important global regulators such as *agr* and *sarA* genes are highly expressed in the Rom^R^ clone compared to the parental strain. Particularly, the derepression of these regulators is in agreement with the increased expression of toxins and proteases encoded by *hla*, *psmα1*, *psmβ1*, and *sspB* and the decreased expression of surface proteins like Protein A, SdrA, SdrC, and SdrD. Especially the increased toxin expression is most likely responsible for the increased cytotoxicity (Smagur et al., 2009; Kantyka et al., 2011; Cheung et al., 2014; Sampedro et al., 2014). There are not many cases where loss of gene function is associated with increased virulence. One such example is the deletion of the *rho* gene causing an upregulation of the SaeRS two-component system in *S. aureus* HG001 (Nagel et al., 2018). SaeRS two-component system acts as a major regulator of virulence gene expression in staphylococci (Liu et al., 2016).

## Conclusion

A model for the FarE mediated resistance to Rom in the Rom^R^ clone is illustrated in Figure 6. We assume that the high Rom resistance is mediated by the overexpression of FarE leading to an increased release of lipids and FAs into the supernatant by which Rom’s antimicrobial activity could be neutralized. The increased cytotoxicity of the Rom^R^ clone is also reflected by its increased virulence in a mouse pneumonia model. In our case inactivation of *farR* gene causes upregulation of the *agr* and *sarA* regulator systems which most likely is responsible for the increased virulence.

## Ethics Statement

All of the animal studies were approved by the local government of Franconia, Germany (approval number 55.2-2532-2-155) and performed in strict accordance with the guidelines for animal care and experimentation of German Animal Protection Law and the DIRECTIVE 2010/63/EU of the EU. The mice were housed in individually ventilated cages under normal diet in groups of four to five throughout the experiment with *ad libitum* access to food and water.

## Author Contributions

FG and M-TN designed the study. M-TN, JoS, PT, MN, PF, SD, JaS, CS, BB, PE, ToH, NK, ThH, DW, KO, SV, and UM performed all experiments. FG and M-TN wrote the manuscript.

## Conflict of Interest Statement

The authors declare that the research was conducted in the absence of any commercial or financial relationships that could be construed as a potential conflict of interest.

## Footnotes

1 https://www.ebi.ac.uk/arrayexpress/

## Abbreviations

Agr: accessory global regulator
COG: cluster of orthologous groups
DMSZ: Deutsche Sammlung von Mikroorganismen und Zellkulturen GmbH
FarE: effector of fatty acid resistance
FarR: regulator of fatty acid resistance
FAs: fatty acids
MBC: minimal bactericidal concentration
MIC: minimal inhibitory concentration
MOA: mode of action
MRSA: methicillin (multiple) resistant *Staphylococcus aureus*
Rom: Rhodomyrtone
SarA: staphylococcal accessory regulator.
