# Supplementary material for: Inactivation of farR Causes High Rhodomyrtone Resistance and Increased Pathogenicity in Staphylococcus aureus
Source: Front Microbiol. 2019 May 28;10:1157. doi: 10.3389/fmicb.2019.01157 (PMC6547885; doi:10.3389/fmicb.2019.01157)
Supplement: Supplementary file 1 [file Data_Sheet_1.PDF]

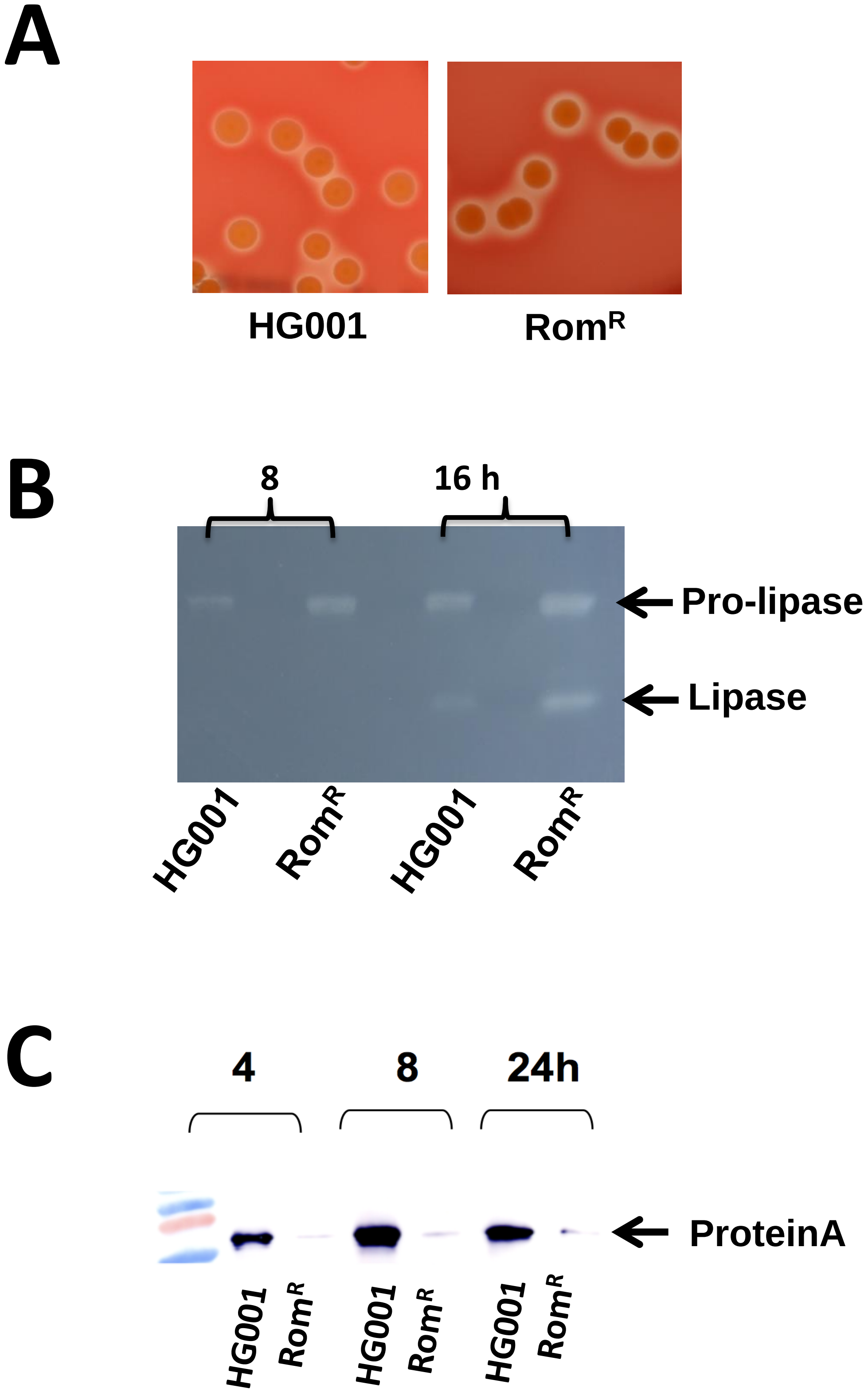

Fig. S1. Phenotypic expression of hemolysin, lipase and protein A in HG001 and the Rom^R clone. (**A**) Hemolytic activity on sheep blood agar; strains were grow for 24 h. The halo around the colonies shows essentially alpha hemolytic activity due to lysed erythrocytes. (**B**) Lipase zymogram of culture supernatants, the strains were grown for 8 and 16 h in TSB. (**C**) Western blot of Protein A, the strains were grown for 8, 16 and 24 h in TSB.

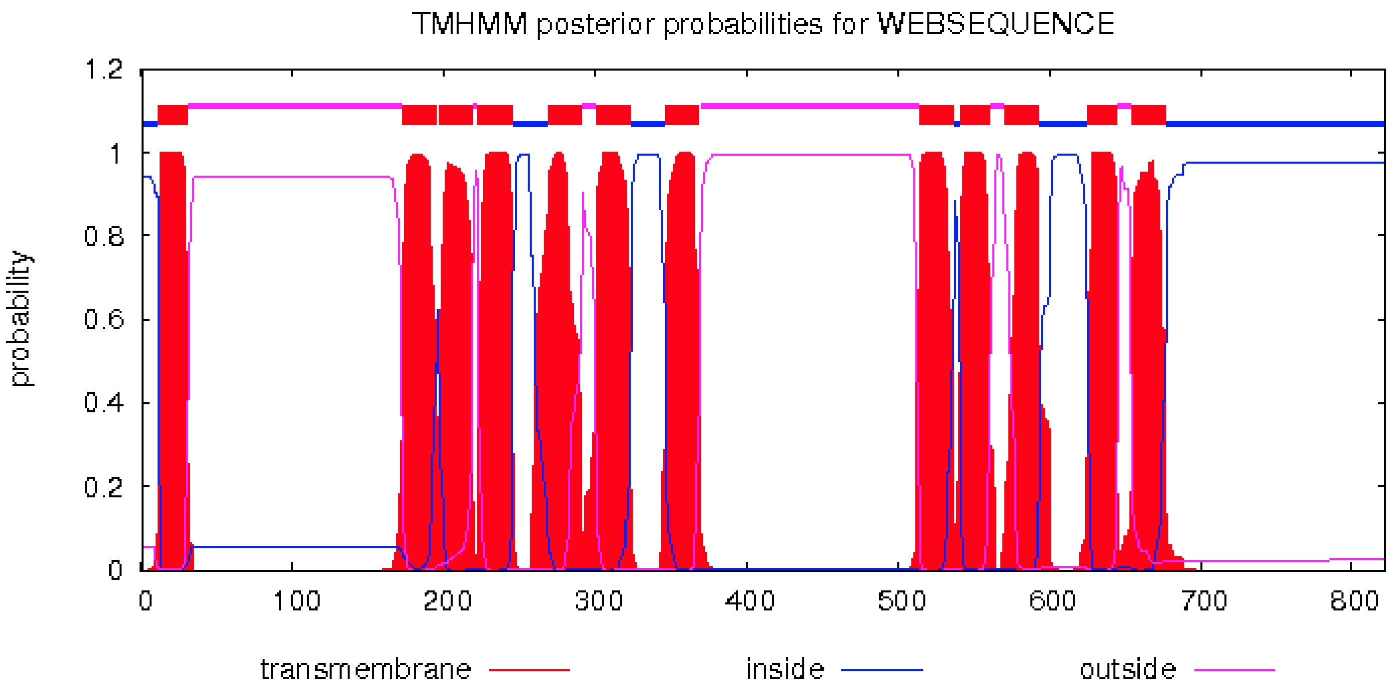

Fig. S2. FarE transmembrane domain prediction. According to the prediction of transmembrane helices software (TMHMM Server v.2.0), FarE is supposed to be a membrane protein with 12 transmembrane domains.

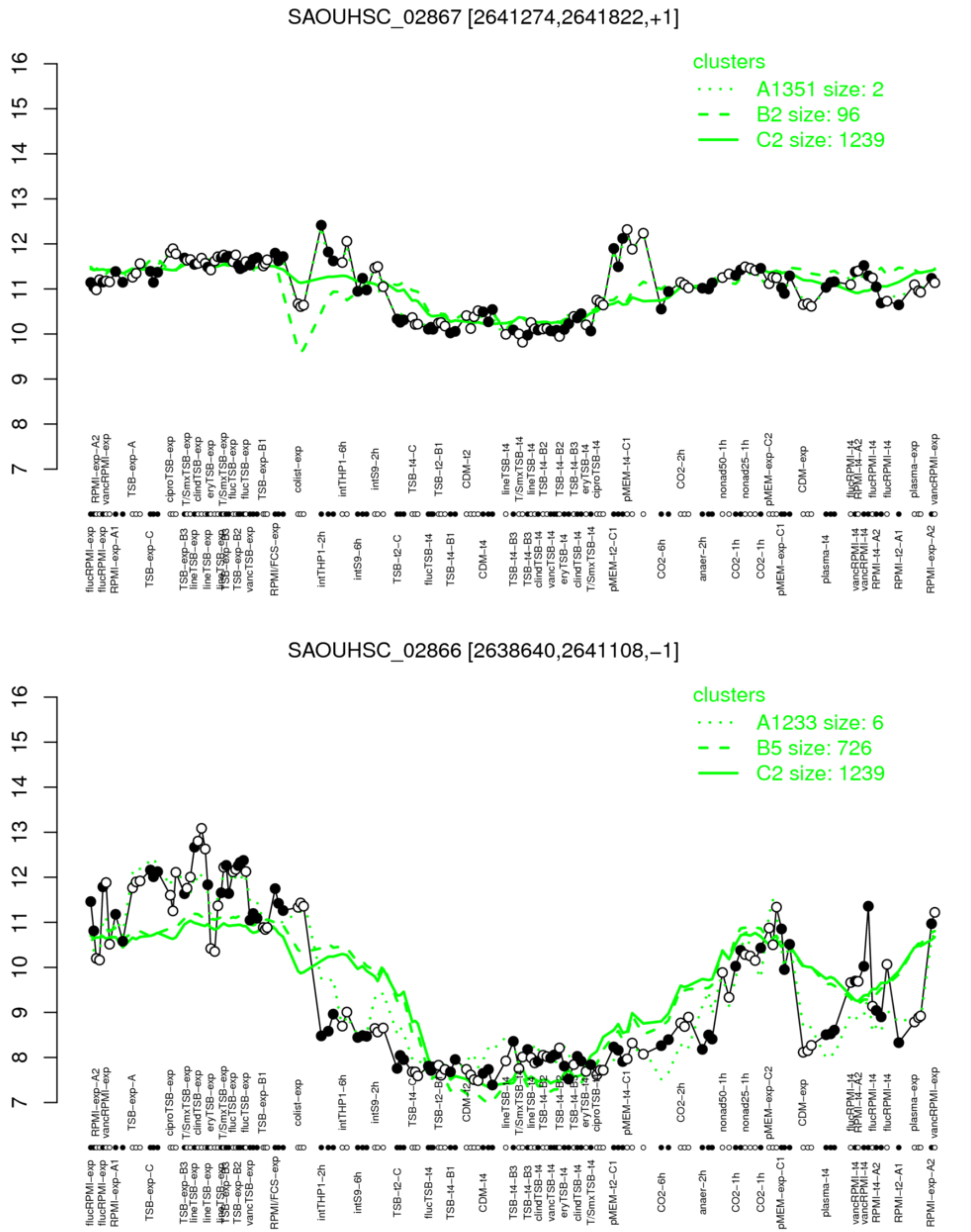

Fig. S3. Expression patterns of *farR* and *farE* of *S. aureus* HG001 under various experimental conditions. The transcriptome of *S. aureus* was analyzed by strand-specific tiling arrays (Mäder et al., 2016). Graphs of condition-dependent gene expression levels were obtained from the Expression Data Browser at http://genome.jouy.inra.fr/cgi-bin/aeb/index.py. The gene-specific pages of the Expression Data Browser, which can be retrieved using the "Locus Tag" field, show additional information including the respective chromosomal region with transcription profiles, mapped transcription units and newly identified RNA features.

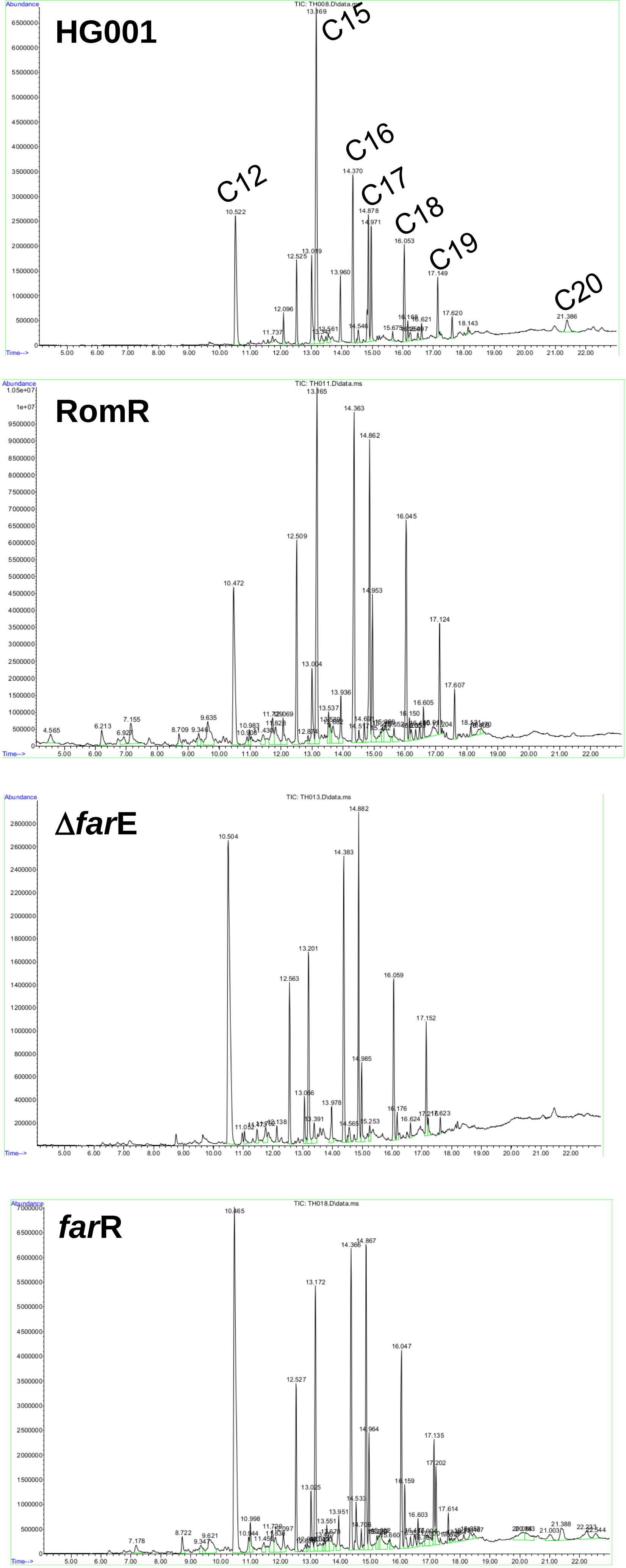

Fig. S4. GC analysis of the fatty acids isolated by total lipid extraction from the supernatant of different *S. aureus* strains after 16 h culture in TSB. This is the representative of two independent experiments. 0.2 mg/ml C12 FA was used as internal standard.
