# Supplementary material for: Inactivation of farR Causes High Rhodomyrtone Resistance and Increased Pathogenicity in Staphylococcus aureus
Source: Front Microbiol. 2019 May 28;10:1157. doi: 10.3389/fmicb.2019.01157 (PMC6547885; doi:10.3389/fmicb.2019.01157)
Supplement: Supplementary file 3 [file Table_2.DOCX]

**Supplementary Tables**

**Table S1. Normalized expression levels and ratios revealed by comparative transcriptome analysis (RNA-seq) of the Rom^R^ clone vs HG001** (Excel file)

**Table S2. Oligonucleotides used in this study**

| Name | Sequence (5-3) |
| --- | --- |
| F_*farE*up | cactcatcgcagtgcagcggACGCTAAAACAGGTAGTC |
| R_*farE*up | gcaaaaaacctccATATACAGTGTAGATTATTGTTCG |
| F_*farE*down | cactgtatatGGAGGTTTTTTGCATGGAAG |
| R_*farE*down | gcccgggtaccgagctccggCGTAAACTCGACAATTTATTTACAAC |
| F_farR (BamHI) | aattaGGATCCAGGAGGTattaat**atg**aaagagactgatt |
| R_farR (XmaI) | tataaCCCGGGAAGCTTActatttaatcttaatattgatt |
